# Selected mechanical properties of human cancellous bone subjected to different treatments: short-term immersion in physiological saline and acetone treatment with subsequent immersion in physiological saline

**DOI:** 10.1186/s13018-022-03265-4

**Published:** 2022-08-06

**Authors:** Fangxing Wang, Florian Metzner, Leyu Zheng, Georg Osterhoff, Stefan Schleifenbaum

**Affiliations:** 1 ZESBO - Center for Research on Musculoskeletal Systems, Department of Orthopedic Surgery, Traumatology and Plastic Surgery, Leipzig University, Semmelweisstraße 14, 04103 Leipzig, Germany; 2 Department of Orthopedic Surgery, Traumatology and Plastic Surgery, Leipzig University, Liebigstraße. 20 Haus 4, 04103 Leipzig, Germany

**Keywords:** Cancellous bone, Mechanical properties, Physiological saline, Acetone, Bone marrow

## Abstract

**Background:**

Physiological saline (0.9% NaCl) and acetone are extensively used for storage (as well as hydration) and removal of bone marrow, respectively, of cancellous bone during preparation and mechanical testing. Our study aimed to investigate the mechanical properties of cancellous bone subjected to short-term immersion in saline and acetone treatment with subsequent immersion in saline.

**Methods:**

Cylindrical samples (Ø6 × 12 mm) were harvested from three positions (left, middle, and right) of 1 thoracic vertebral body, 19 lumbar vertebral bodies, and 5 sacral bones, as well as from 9 femoral heads. All samples were divided into two groups according to the different treatments, (i) samples from the left and middle sides were immersed in saline at 4℃ for 43 h (saline-immersed group, *n* = 48); (ii) samples from the respective right side were treated with a combination of acetone and ultrasonic bath (4 h), air-dried at room temperature (21℃, 15 h), and then immersed in saline at room temperature (21℃, 24 h) (acetone and saline-treated group, *n* = 38). All samples were subjected, both before and after treatment, to a non-destructive compression test with a strain of 0.45%, and finally destructive tests with a strain of 50%. Actual density (*ρ*_act_), initial modulus (*E*_0_), maximum stress (*σ*_max_), energy absorption (*W*), and plateau stress (*σ*_p_) were calculated as evaluation indicators.

**Results:**

Based on visual observation, a combination of acetone and ultrasonic bath for 4 h failed to completely remove bone marrow from cancellous bone samples. The mean values of *ρ*_act_, *σ*_max_, *W*, and *σ*_p_ were significantly higher in the femoral head than in the spine. There was no significant difference in *E*_0_ between non-treated and saline-immersed samples (non-treated 63.98 ± 20.23 vs. saline-immersed 66.29 ± 20.61, *p* = 0.132). The average *E*_0_ of acetone and saline-treated samples was significantly higher than that of non-treated ones (non-treated 62.17 ± 21.08 vs. acetone and saline-treated 74.97 ± 23.98, *p* = 0.043).

**Conclusion:**

Short-term storage in physiological saline is an appropriate choice and has no effect on the *E*_0_ of cancellous bone. Treatment of cancellous bone with acetone resulted in changes in mechanical properties that could not be reversed by subsequent immersion in physiological saline.

**Supplementary Information:**

The online version contains supplementary material available at 10.1186/s13018-022-03265-4.

## Background

Knowledge of the mechanical properties of cancellous bone is beneficial not only for understanding degenerative bone diseases such as osteoporosis and osteoarthritis [1–3], but also for the development of bone grafting techniques and substitute materials, as well as their clinical application in trauma and orthopaedic surgery [3–5].

Experimental studies addressing the mechanical properties of cancellous bone are often conducted on the cadaveric bone to reflect in vivo performance. To simulate in vivo conditions, bone samples are typically immersed in physiological saline during preparation and mechanical testing [6–9]. One question arises, whether the mechanical properties of cancellous bone are affected by storage in physiological saline. Previous reports on this subject have mainly concentrated on cortical bone [10, 11]. However, cancellous bone differs significantly from cortical bone in terms of compositional characteristics, microstructure, and mechanical performance [1, 12]. The influence of physiological saline storage on the mechanical properties of cancellous bone may differ from that of cortical bone. To date, it remains unclear the length of time that cancellous bone can be stored in physiological saline without affecting the mechanical properties.

Apart from that, several studies have reported that the presence of bone marrow has a significant effect on the mechanical properties of cancellous bone [6, 8, 9]. Thus, mechanical properties will be variable depending on whether the samples are tested with or without marrow. Of which, the chemical composition of yellow marrow is 80% lipid, 15% water, and 5% protein, and the fat content increases with age [13]. Consequently, acetone, as a classic defatting solvent, has been commonly used in the laboratory to remove bone marrow from cancellous bone samples [14–16]. Another question arises, whether there are any differences between the mechanical properties of non-treated bone and bone subjected to immersion in physiological saline after treatment with acetone. On this topic, Fischer et al. [17] found that there was a significant difference in mechanical properties between non-treated bone samples and those treated with acetone. However, their study did not immerse the acetone-treated samples anymore. Hence, it was unclear if the changes in mechanical properties after acetone treatment could be reversed by subsequent immersion in physiological saline. Moreover, current studies investigating the influence of bone marrow removal on the mechanical properties of cancellous bone are few and mostly limited to animal bones [8] or human bone from a single anatomical site [17]. Due to the varying loads, the density and microstructure of human cancellous bone differ from those of animals, resulting in some variability in mechanical properties [18]. Meanwhile, human cancellous bone exhibits a high variance in mechanical properties due to the different anatomic sites [19, 20].

To address these issues, the given experimental study aimed at investigating the mechanical properties of human cancellous bone subjected to different treatments, i.e., short-term immersion in physiological saline and acetone treatment with subsequent immersion in physiological saline.

## Methods

### Donors

Donor bones were collected within a maximum of 24 h post-mortem, and then stored at − 80℃ (fresh and anatomically unfixed condition). All bones were harvested from given donors through the same procedure. Eventually, 1 thoracic vertebral body (T_12_), 19 lumbar vertebral bodies (L_1–5_), 5 sacral bones (S_1–5_), and 9 femoral heads were obtained from 5 donors without fractures (based on CT-images), with an average age of 81 years (range: 65–91 years, 3 males and 2 females).

Moreover, all body donors had given their informed and written consent to the donation of their bodies for teaching and research purposes while alive. Being part of the body donor program is regulated by the Saxonian Death and Funeral Act of 1994 §18 (8), institutional approval for the use of post-mortem tissues from human body donors was obtained from the Institute of Anatomy of our University. The authors declare that all experiments were performed in accordance with the principles of the Declaration of Helsinki.

### Sample preparation

**Fig. 1:**
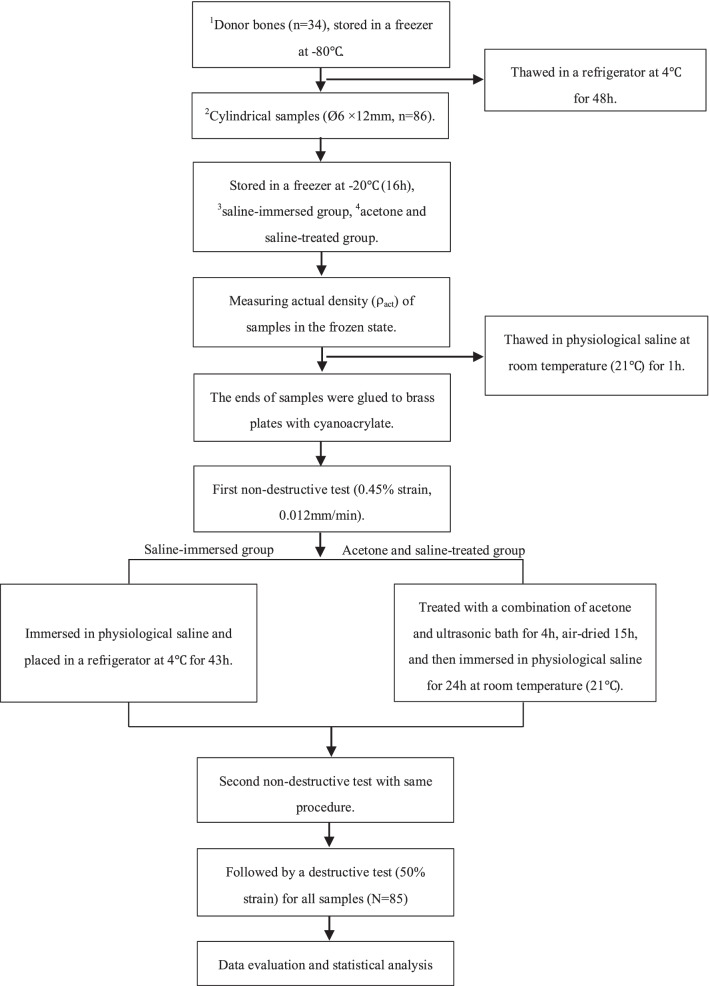
The flow chart of the main steps in our experiment. ^1^Human donors (*n* = 34) included 1 thoracic vertebral body (T_12_), 19 lumbar vertebral bodies (L_1–5_), 5 sacral bones (S_1–5_), and 9 femoral heads. ^2^Cylindrical samples (Ø6 × 12 mm, *n* = 86) were obtained using a tenon cutter and a stationary drilling machine, including 63 samples obtained from spinal vertebral bodies and 23 samples collected from femoral heads. ^3^Saline-immersed group: the samples from the left and middle sides of spinal vertebral bodies and femoral heads. ^2^Acetone and saline-treated group: the samples from the respective right side of spinal vertebral bodies and femoral heads

The harvested donor bones were stored in a freezer at − 80℃ for 5 years (range: 4–6 years) and thawed in a refrigerator at 4℃ for 48 h prior to mechanical testing. The main steps of our experiment were presented in the form of a flow chart (Fig. 1).

#### Femoral heads

**Fig. 2:**
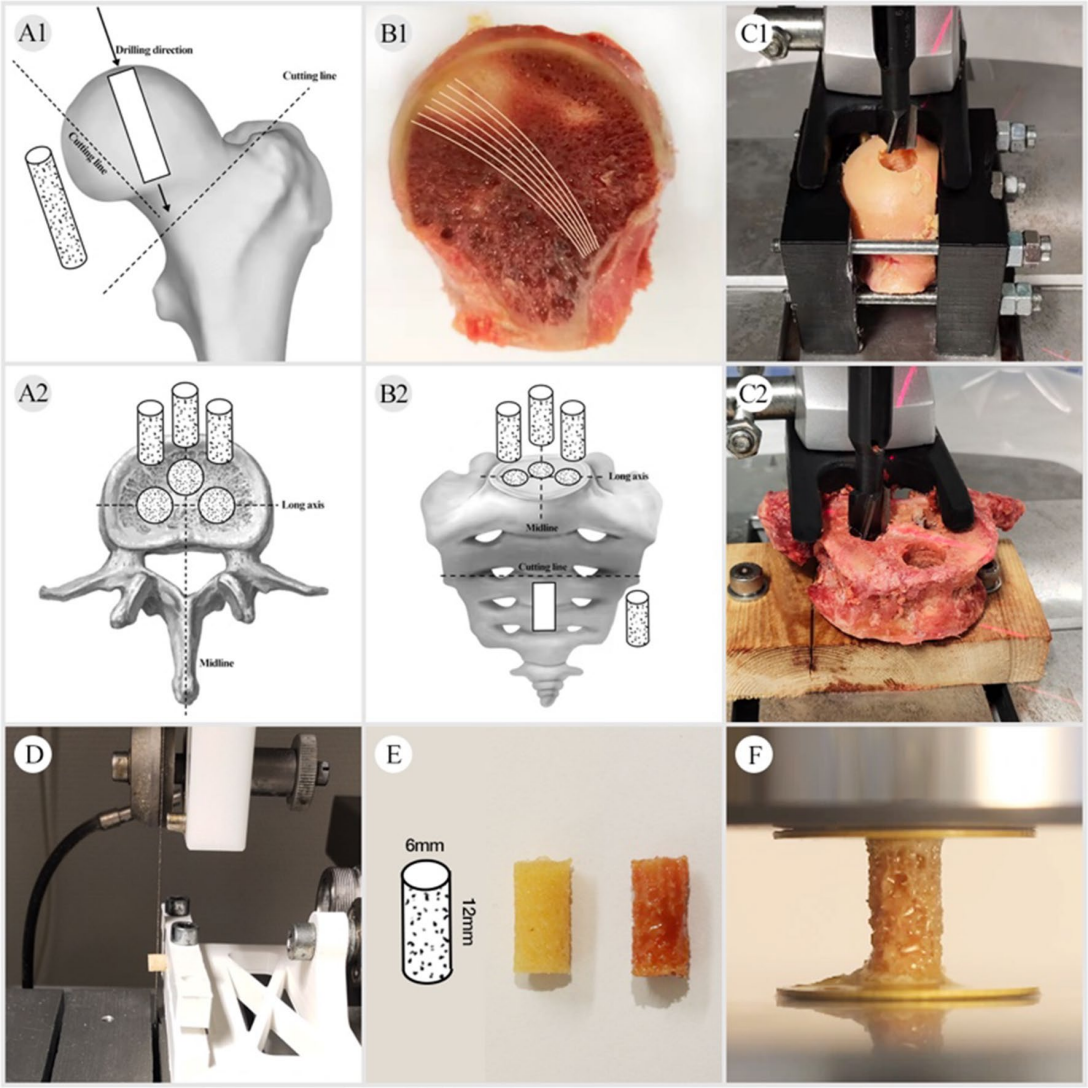
Sample preparation: **A1** shows the location of the femoral head samples within the available region. **B1** The main trabecular orientation was visible after cutting. **C1** The femoral head was fixed in the clamping jaws and then drilled in the superior-inferior direction along with the main trabecular orientation. **A2**–**C2** Cylindrical cores were harvested from three positions (left, middle, and right) of 1 thoracic vertebral body (T_12_), 19 lumbar vertebral bodies (L_1–5_), and 5 sacral bones (S_1–5_) along the long and midline axis. **D**, **E** The cores were cut into samples (Ø6 × 12 mm). **F** The samples were tested after being glued to the platens with cyanoacrylate

Cylindrical cores (Ø6 mm) were collected from femoral heads using a tenon cutter and a stationary drilling machine in the superior-inferior direction along with main trabecular orientation (Fig. 2A1–C1), as previously described [21]. The proximal and distal ends of each core were marked separately. Finally, the cylindrical cores were fixed in 3D-printed jaws to be cut with a diamond saw to obtain the samples to be tested (Fig. 2D–E).

#### Spine

Cylindrical cores (Ø6 mm) were harvested from three positions (left, middle, and right) of 1 thoracic vertebral body (T_12_), 19 lumbar vertebral bodies (L_1–5_), 5 sacral bones (S_1–5_) along the long and midline axis using a tenon cutter and a stationary drilling machine (Fig. 2A2–C2). Similarly, the proximal and distal ends of the cylindrical cores were marked and cut with a diamond saw to obtain the samples to be tested.

In this study, a sample geometry of Ø6 × 12 mm was used. As suggested by Keaveny TM et al. [22], a 2:1 cylinder with a minimum diameter of 5 mm would be sufficient to satisfy the continuum assumption. Also, cylindrical samples with a diameter of 6 mm were adopted in our study to ensure that they could be obtained from all anatomical sites, as the size of lumbar vertebrae varies with height and gender.

To standardize the measurement criteria and reduce deviations caused by the fluid flow of bone marrow, the weight (*m*_air_) of each sample was measured with a precision scale in the frozen state. The diameter and length of each sample were measured with a caliper (accuracy 0.02 mm) to calculate a total volume (*V*_0_). Based on these test parameters, the actual density (*ρ*_act_) [23] of samples was calculated as follows:

1 $${\text{Total}}\;{\text{volume}}:\;v_{0} = \pi \left( {d{/}2} \right)^{2} l$$

2 $${\text{Actual}}\;{\text{density}}:\;\rho_{{{\text{act}}}} = {\raise0.7ex\hbox{${m_{{{\text{air}}}} }$} \!\mathord{\left/ {\vphantom {{m_{{{\text{air}}}} } {v_{0} }}}\right.\kern-\nulldelimiterspace} \!\lower0.7ex\hbox{${v_{0} }$}}$$

### Group assignment and treatments

In total, 86 samples (spinal vertebrae, *n* = 63 vs. femoral head, *n* = 23) were harvested, and then divided into two groups, saline-immersed group (*n* = 48), as well as acetone and saline-treated group (*n* = 38). Samples in the saline-immersed group were from the left (*n* = 18) and middle (*n* = 21) sides of spinal vertebral bodies, as well as the left femoral heads (*n* = 9). Samples in the acetone and saline-treated group were from the respective right side (Fig. 2). All samples were wrapped in plastic foil and stored in a freezer at − 20℃ for 16 h depending on the working time regulations of our laboratory. Then, all frozen samples were thawed in physiological saline at room temperature (21℃) for 1 h [24] after completing density measurements.

The samples in the saline-immersed group were immersed in physiological saline and placed in a refrigerator at 4℃ for 43 h. Prior to testing, samples were removed from the 4℃ refrigerator and placed at room temperature (21℃), followed by mechanical testing. The samples in the acetone and saline-treated group were subjected to a combination of acetone and ultrasonic bath for 4 h, the main parameters of ultrasonic cleaner are shown in Table 1. The water within the tank was replaced once at the end of a cycle (20 min) to avoid damaging the mechanical properties of samples due to excessive water temperature [14]. After treatment, all samples were air-dried at room temperature (21℃) for 15 h, followed by immersion in physiological saline for 24 h at room temperature (21℃) [25–27].

**Table 1:** The main data of materials and equipment used in the present study

Material and equipment	Manufacturer and/or batch number	Model and/or parameter
Physiological saline	Fresenius Kabi AG, Bad Homburg, Germany. 13QIP251	0.9% NaCl
Acetone	Carl Roth GmbH + Co.KG, Karlsruhe, Germany. 2006622	Purity ≥ 99.7%, concentration ≥ 99.5%, molar mass: 58.08 g/mol, density: 0.79 g/ml
Ultrasonic cleaner	Gute ultrasonic Co., Ltd, Guangdong, China	VGT-1730QTD. Ultrasonic power 100 W, ultrasonic frequency: 40 kHz, heating power 100 W
Tenon cutter	FAMAG-Werkzeugfabrik GmbH & Co. KG, Remscheid, Germany	FAMAG Series 1616
Stationary drilling machine	Robert Bosch GmbH Power Tools, Leinfelden-Echterdinge, Germany	PBD 40
Diamond saw	EXAKT Advanced Technologies. GmbH, Norderstedt, Germany	EXAKT 310
Precision scale	Mettler-Toledo GmbH, Gießen, Germany	ML303T/00
Digital camera	Canon INC. Tokyo, Japan	Canon EOS 70D
Servo-electric single-axis testing machine	Zwick/Roell, Ulm, Germany	Allroundline Z10

Before and after treatment, samples were photographed with a digital camera. The clearer the pores and the whiter the color of the sample, the more effective the treatment proved to be.

### Mechanical testing

Uniaxial compression tests were performed using a servo-electric single-axis testing machine equipped with a 2.5 kN force transducer at room temperature (21℃) (Fig. 2F). In order to eliminate the end-artifacts, the ends of samples were glued to brass plates with cyanoacrylate [28]. Of which, samples in the acetone and saline-treated group need to be glued twice before and after treatment due to the dissolution effect of acetone on cyanoacrylate. All samples were subjected, both before and after treatment, to a non-destructive compression test with a strain of 0.45%, and finally a destructive test with a strain of 50%. The samples were stored in physiological saline (at room temperature, 21℃) during the process of mechanical testing (within a maximum of 30 min) to prevent drying.

**Fig. 3:**
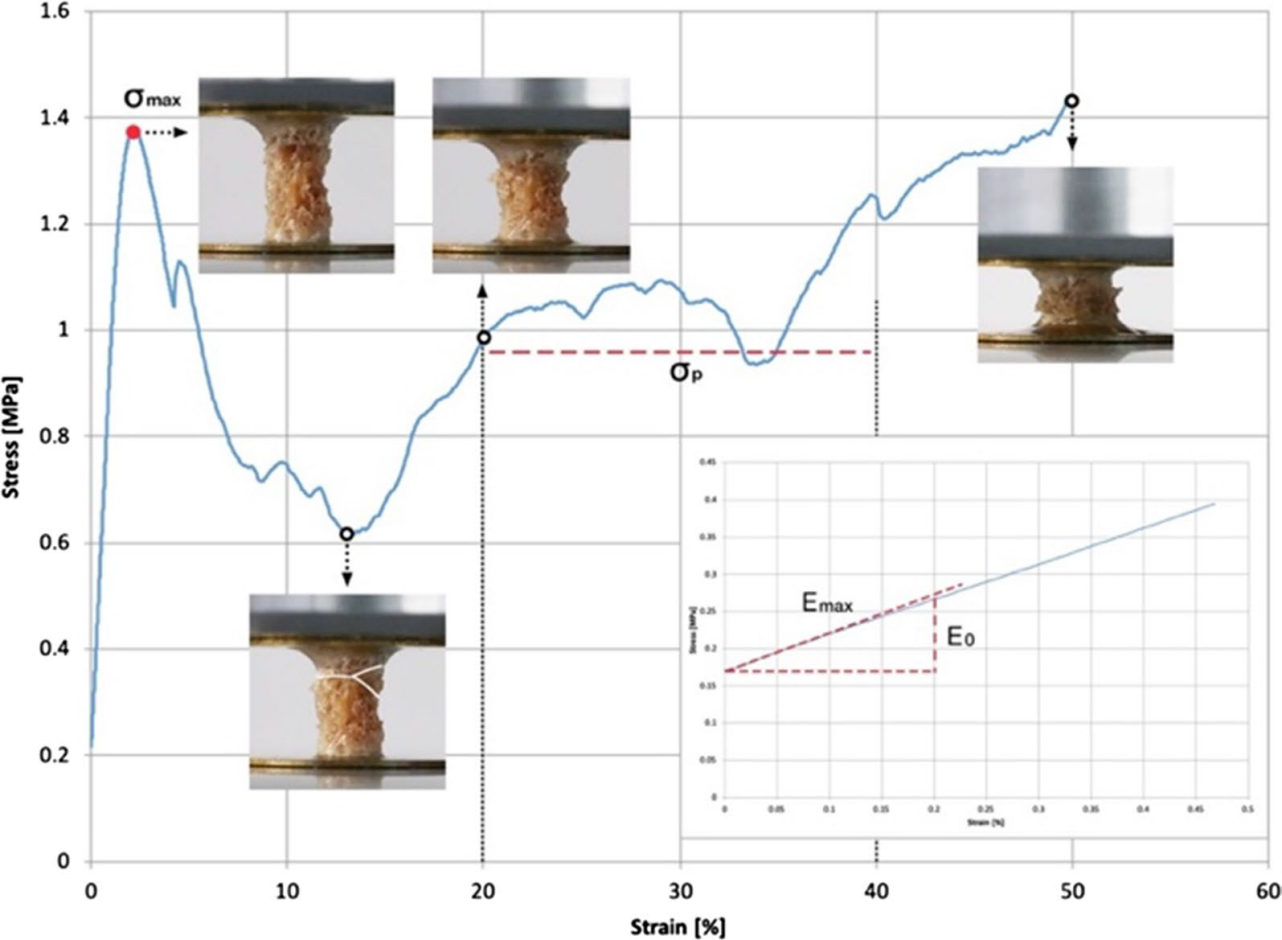
Typical non-destructive and destructive stress–strain curves of a cancellous sample plotted with the evaluation of deformed shape. *E*_0_: initial modulus (0–0.2% strain, MPa), *E*_max_: maximum modulus (0–0.2% strain, MPa), *σ*_max_: maximum stress (MPa), *σ*_p_: plateau stress (20%-40% strain, MPa)

The non-destructive testing procedure started with the application of a preload of 5 N where zero strain was defined. The samples reached 0.45% strain at a constant deformation rate of 0.012 mm/min [6]. Destructive testing was conducted as described by Metzner et al. [21] with the following changes. Testing speed was set to 0.012 mm/s for the first 10% strain and a follow-up speed of 0.12 mm/s. No hysteresis loop was conducted as the modulus was calculated during non-destructive testing. The test was stopped at 50% strain, and then maximum stress (*σ*_max_) and plateau stress (*σ*_p_) were calculated. Figure 3 presents typical non-destructive and destructive stress–strain curves. It is discussed in terms of stress (axial compressive load divided by the apparent cross section area) against the strain (displacement divided by the initial length). In addition, Table 1 describes the main data of the materials and equipment used in the present study.

### Data evaluation and statistical analysis

The test data were computed and analyzed using textXpert III (ZwickRoell GmbH & Co KG, Ulm, Germany), MATLAB 2011 (MathWorks, Natick, MA, USA), and Excel 2013 (Microsoft Corporation, Redmond, WA, USA). The initial modulus (*E*_0_), defined as the slope at zero strain of a second-order polynomial fit to the portion of the stress–strain curve from 0–0.2% strain [29], was compared with the maximum modulus (*E*_max_) calculated by the maximum slope of the stress–strain curve from 0–0.2% strain (Fig. 3). If the difference between *E*_0_ and *E*_max_ was more than 5%, the sample was not included in the evaluation. This excluded samples with a "toe-region" in the stress–strain curve, which is an indication of an end-artifact.

Statistical comparison of the data was performed using the SPSS 23.0 (Statistical Package for Social Science, IBM, USA). The *Shapiro–Wilk test* was used to determine the distribution of the data. If the variables were normally distributed, the *Student’s t-test* was used for data sets, and either *independent or paired t-tests* were employed depending on the samples being from between or within groups, respectively. Otherwise, the *Wilcoxon-test* or *Mann–Whitney U test* was used for data sets, depending on paired or independent samples. Consecutive data were summarized as mean ± standard deviation ($\overline{X}$ ± SD). Statistical significance was set at *p* < 0.05.

## Results

The most of cylindrical cores (*n* = 45) from the spinal vertebrae were approximately 15–20 mm in length, and only one sample (Ø6 × 12 mm) was obtained from each core. There were 9 cores, each of which can obtain 2 samples due to longer lengths (about 26 mm). Cylindrical cores (*n* = 8) from the femoral heads were about 42 mm in length. So, three samples can be obtained from one cylindrical core. Of these, one sample was damaged during the cutting. Finally, 86 samples (spinal vertebrae, *n* = 63 vs. femoral head, *n* = 23) were subjected to non-destructive testing, of which 85 were included in the destructive testing as one fractured before testing.

A total of 18 samples (spinal vertebrae, *n* = 5 vs. femoral heads, *n* = 13) were excluded since the difference between *E*_0_ and *E*_max_ was more than 5%. Additionally, 15 samples (spinal vertebrae, *n* = 12 vs. femoral heads, *n* = 3) had to be excluded due to other methodologic errors. This was done to exclude any samples with experimental artifacts leading to an underestimation of mechanical properties, which referred to previous research [30, 31]. For the saline-immersed group, 31 samples were from spinal vertebral bodies (left site, *n* = 12 vs. middle side, *n* = 19), and 4 samples were from left femoral heads. For the acetone and saline-treated group, 15 samples were from the right site of spinal vertebral bodies, and 3 samples were from left femoral heads. Finally, 53 samples (saline-immersed group, *n* = 35 vs. acetone and saline-treated group, *n* = 18) were included. More detailed data could be available in the Additional files 1 and 2.

### Bone marrow removal efficiency

**Fig. 4:**
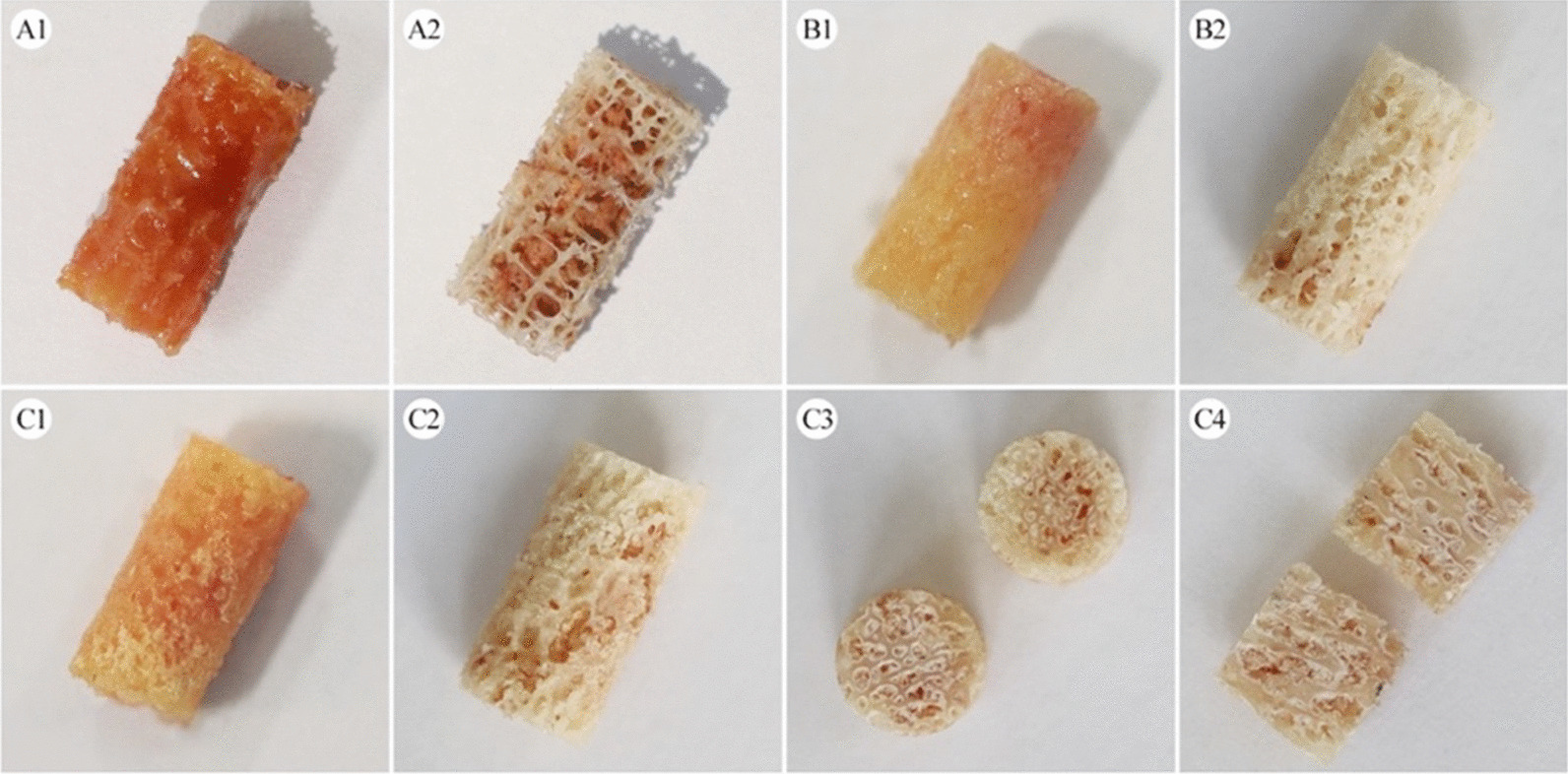
The appearance of non-treated, as well as acetone and saline-treated samples. **A1**–**A2** the low-density sacral sample before and after treatment by a combination of acetone and ultrasonic bath (4 h); **B1**–**B2**/**C1**–**C2**: the relatively dense femoral head samples before and after treatment by a combination of acetone and ultrasonic bath (4 h); **C3**–**C4**: one of the samples (*n* = 3) was split longitudinally and vertically through the center. The samples treated with a combination of ultrasonic bath and acetone (4 h) still had a certain amount of bone marrow left

After being treated with a combination of acetone and ultrasonic bath for 4 h, the samples still contained a certain amount of bone marrow based on visual observation. Furthermore, three samples from one cylindrical core were split longitudinally and vertically through the center, the remaining lipids in the deeper pores could be visualized more clearly (Fig. 4). Hence, we could tentatively assume that a combination of acetone and ultrasonic bath for 4 h failed to completely remove bone marrow from cancellous bone samples (Ø6 × 12 mm), not only in relatively dense femoral samples, but also in low-density lumbar and sacral bone samples. Nevertheless, our findings still need to be further quantitative analyzed in future.

### Distribution of actual density and initial modulus

**Fig. 5:**
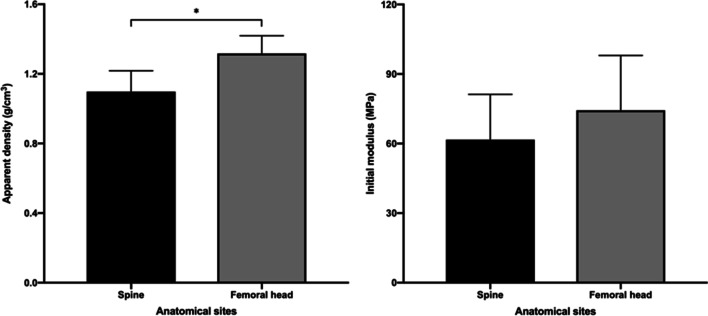
The distribution of actual density (*ρ*_act_, g/cm^3^) and initial modulus (*E*_0_, MPa) in the spine (thoracic, lumbar vertebral bodies, and sacral bones) and femoral heads (*p < 0.05)

In the present study, the *ρ*_act_ of samples obtained from the femoral head was significantly higher than that of the spine (femoral head, 1.32 ± 0.10 g/cm^3^ vs. spine, 1.10 ± 0.12 g/cm^3^ *p* = 0.000). The average *E*_0_ of samples obtained from the femoral head was higher than that obtained from the spine (femoral head 74.37 ± 23.62 MPa vs. spine 61.69 ± 19.54 MPa, *p* = 0.126) (Fig. 5).

### Saline and acetone groups

**Fig. 6:**
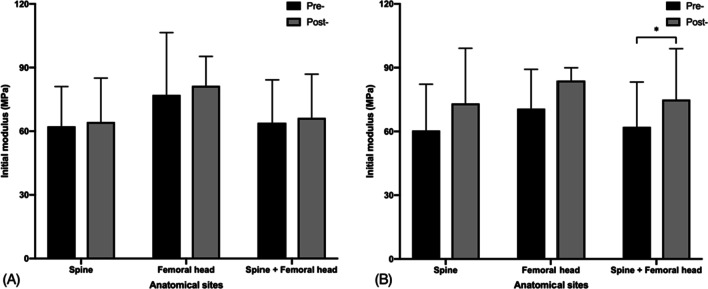
The initial modulus (*E*_0_, MPa) in different groups before and after treatment: **A** saline-immersed group, **B** acetone and saline-treated group (*p < 0.05)

In the saline group, there was no significant difference in the *E*_0_ between non-treated and saline-immersed samples (non-treated 63.98 ± 20.23 vs. saline-immersed 66.29 ± 20.61, *p* = 0.132). In the acetone and saline-treated group, the average *E*_0_ was significantly higher than that of non-treated ones (non-treated 62.17 ± 21.08 vs. acetone and saline-treated 74.97 ± 23.98, *p* = 0.043) (Fig. 6).

**Fig. 7:**
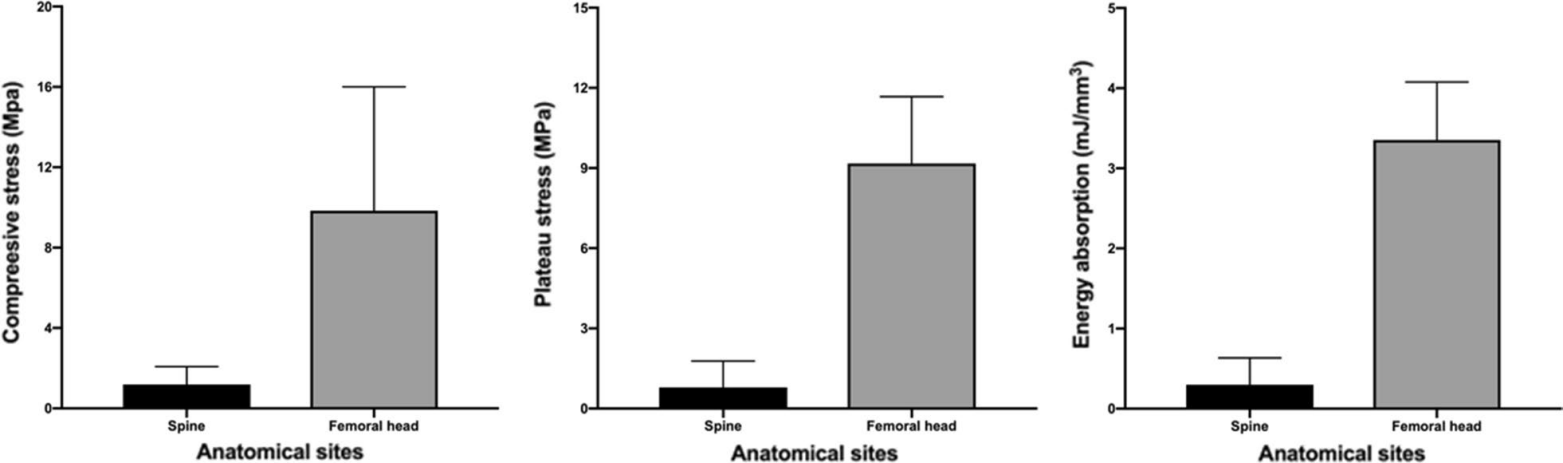
From destructive testing, femoral head has higher mechanical properties than spine in several parameters, including maximum stress (*σ*_max_, MPa), plateau stress (*σ*_p_, MPa), and energy absorption (*W*, mJ/mm^3^)

The results of destructive tests on samples from the saline group (*n* = 35) indicated that the femoral head has higher mechanical properties than the spinal bone in several parameters, including *σ*_max_ (femoral head 9.84 ± 6.16 vs. spine 1.19 ± 0.89), *σ*_p_ (femoral head 9.18 ± 2.50 vs. spine 0.79 ± 0.99), and *W* (femoral head 3.35 ± 0.72 vs. spine 0.30 ± 0.33) (Fig. 7).

## Discussion

The aim of the study was to investigate the effects of two treatments, short-term immersion in physiological saline and acetone treatment with subsequent immersion in physiological saline, on the mechanical properties of cancellous bone. Cancellous bone samples from multiple anatomic sites were subjected to a non-destructive compression test before and after treatment, followed by a self-control analysis. This is due to the fact that even if the samples are from the same anatomic site, variations in microstructure and mechanical properties still exist between different locations [32]. To our knowledge, this is the first study to report the appropriate time for storage of human cancellous bone in physiological saline and the effect of acetone treatment with subsequent immersion in physiological saline on mechanical properties, as well as the efficiency of bone marrow removal by a combination of acetone and ultrasonic bath for cancellous bone samples from multiple anatomical sites.

Regarding the influence of saline storage on the mechanical properties of bone tissue, Linde and Sørensen [6] found that fresh trabecular bone stored in physiological saline for 24 h experienced a 10% decrease in stiffness and unloading energy. But they attributed this difference to post-mortem changes in bone tissue and did not consider it to be an effect of storage in saline. In addition, Gustafson et al. [10] reported that calcium ions would dissolve from bone specimens (cortical portion in the third metacarpal of equine) maintained for 6 days in a saline bath (0.9% NaCl) at room temperature. The loss of minerals significantly decreased the elastic modulus of the bone (approximately 2.4%), whereas the modulus of the bone specimens did not change significantly when immersed in calcium-buffered saline. However, the experimental material they employed was animal cortical bone rather than human cancellous bone, both of which have significant differences in structural and mechanical characteristics. Moreover, they did not determine the minimum length of time that immersion in saline produced a significant reduction in modulus. The results of the present study suggested that short-term storage (43 h, 4℃) in physiological saline is an appropriate choice with no significant effect on the *E*_0_ of human cancellous bone. Whether prolonged storage in physiological saline affects the mechanical properties needs to be further investigated in future.

Furthermore, the results of our study revealed that treatment of cancellous bone with acetone resulted in changes in mechanical properties that could not be reversed by subsequent immersion in physiological saline. A significant increase in the *E*_0_ was observed after acetone treatment with subsequent immersion in physiological saline. In line with our results, a study by Fischer et al. [17] reported that the mean value of elastic modulus was higher for samples stored in acetone for 24 h than that of non-treated samples, the increase reached 146%. They attributed this difference to the defatting of cancellous bone by acetone. By comparison, the average *E*_0_ increase was only 21% (non-treated samples: 62.17 MPa vs. acetone and saline-treated samples: 74.97 MPa) in the present study. Apart from the variations in the acetone treatment method and time, more importantly, Fischer’s study did not immerse the acetone-treated samples in physiological saline anymore. Thus, we assumed that this difference mainly originates from whether the samples were immersed with physiological saline or not. Meanwhile, Fischer and co-workers also found that the samples stored in acetone were more brittle than the fresh ones, which provides further support for our assumption. Additionally, concerning the influence of bone marrow on the mechanical properties of cancellous bone. A study by Halgrin J et al. [9] is also in agreement with our results. They reported that the presence of bone marrow would decrease the mechanical properties of trabecular bone (26% for elastic modulus, 38% for maximum compressive stress, and 33% for average stress). Therefore, in combination with our results, it could be concluded that the removal of bone marrow increases the *E*_0_ of cancellous bone in an unconfined compression test and cannot be compensated by physiological saline immersion, which may be related to the difference in viscosity properties of both [33, 34]. During the deformation, the higher viscosity of bone marrow presents higher resistance to fluid flow, leading to higher stress concentration alone the trabeculae and an earlier breakdown of the trabecular structure, as demonstrated by Halgrin and co-workers [9] using a finite element model.

In addition, the concept of bone marrow removal is relatively simple, but selecting an appropriate and effective method is challenging for cancellous bone. Ultrasonic bath is a process that uses ultrasound (usually from 20–40 kHz) to agitate a fluid, which can accelerate the surface treatment process [35]. It has been widely used in industry for decades, especially for cleaning of small and complex parts. In the laboratory, it is also a common way to remove bone marrow from cancellous bone samples, which has been reported in multiple publications [23, 36, 37]. A combination of ultrasonic bath and trichloroethylene for 4 h is capable of removing all fat in femoral head samples (diameter 9.5 mm, length 5-10 mm), as reported by Sharp et al. [23]. Compared to trichloroethylene, acetone has the advantages of low-cost, easy accessibility, and low toxicity [38]. Moreover, acetone has been proven to be an effective defatting solvent for 2 mm thick cancellous bone slices in our previous study [14]. Hence, in the present study, acetone was chosen as a substitute for trichloroethylene. However, according to the results by visual observation, cancellous bone samples (Ø6 × 12 mm) still have a certain amount of bone marrow residue after being treated with a combination of acetone and ultrasonic bath for 4 h. In comparison with the results of Sharp et al. [23], it can be tentatively concluded that trichloroethylene has a stronger ability of bone marrow removal than acetone. But the severe toxicity of trichloroethylene has to be considered. It can induce cancer in rats and mice and its metabolites are also known to be toxic for the liver, kidney, and lungs [38, 39]. Considering the health of the operator and the growing of green chemistry concept, the search for organic solvents with low toxicity and high efficiency for the removal of marrow from cancellous bone samples needs to be further explored in future.

To minimize the heterogeneity of cancellous bone and to obtain more comprehensive and accurate values. Our study was performed on specimens from multiple anatomical sites of human cancellous bone, including 1 thoracic vertebral body (T_12_), 19 lumbar vertebral bodies (L_1–5_), and 5 sacral bones (S_1–5_), and 9 femoral heads. However, our study also has a few limitations. First, the limitation of the number of specimens, i.e., the study material was harvested from 5 donors. On the one hand, the collection of human cancellous bone is relatively difficult because of the limited availability of donors, as well as the higher requirements for transportation and preservation. On the other hand, some samples were excluded based on our strict inclusion criteria to obtain more accurate experimental results. Second, the samples in the acetone group were glued to the brass plates twice before and after treatment. The small discrepancy between the two operations is likely to produce a deviation. Third, limited by the current experimental conditions, the lipid content and water content of the samples after treatments with acetone and physiological saline were not quantified. Currently, there are still relatively few relevant studies on this subject, which will be the direction of our further research in future. Even considering the limitations mentioned in our experiment, the authors are confident that the results of this study can provide valuable references for the preservation and mechanical testing of cancellous bone. Fourthly, the lack of comprehensive medical data on donor bones in this study is one of the limitations of the results obtained since different types of bone may occur differently when subjected to the same treatment. Lastly, continuous monitoring of storage conditions for bone specimens, together with a notification system for exceeding established acceptance criteria, is a guarantee of reliable and reproducible study results. This issue will be improved and addressed in our future research.

## Conclusion

The mechanical properties of human cancellous bone are very heterogeneous, with the femoral head having significantly higher mechanical properties than the spine. Based on visual observation, cancellous bone samples (Ø6 × 12 mm) still have a certain amount of bone marrow residue after being treated with a combination of acetone and ultrasonic bath for 4 h. When comparing the mechanical properties of cancellous bone with two different treatments, i.e., short-term immersion in physiological saline and acetone treatment with subsequent immersion in physiological saline. We found that short-term storage in physiological saline is an appropriate choice with no significant effect on the *E*_0_ of cancellous bone while acetone increases *E*_0_. Treatment of cancellous bone with acetone resulted in changes in mechanical properties that could not be reversed by subsequent immersion in physiological saline.

## Supplementary Information

**Additional file 1**. Flow chart of the detailed data on the samples tested in our study.

**Additional file 2**. The detailed data of donor bones, cylindrical cores, and samples in our study.

## Data Availability

The data used and/or analyzed during the current study are available from the corresponding author on reasonable request.

## Abbreviations

3D: Three-dimensional
USA: United States of America
MA: Massachusetts
WA: Washington
IBM: International Business Machines Corporation
NaCl: Sodium chloride
CT: Computerized tomography
SPSS: Statistical Package for the Social Sciences
SD: Standard deviation

## Declarations

### Patient and public involvement

No patient involved.
