# Supplementary material for: Selected mechanical properties of human cancellous bone subjected to different treatments: short-term immersion in physiological saline and acetone treatment with subsequent immersion in physiological saline
Source: J Orthop Surg Res. 2022 Aug 6;17:376. doi: 10.1186/s13018-022-03265-4 (PMC9357305; doi:10.1186/s13018-022-03265-4)
Supplement: Supplementary file 1 — Additional file 1. Flow chart of the detailed data on the samples tested in our study. [file 13018_2022_3265_MOESM1_ESM.docx]

Expected cylindrical cores (**n=75**) from left, middle, and right sites.

1 thoracic vertebral body (T_12_), 19 lumbar vertebral bodies (L_1-5_), and 5 sacral bones (S_1-5_). **In total: n=25**.

Femoral heads: left side (n=4), right side (n=5). **In total: n=9**.

Donor (n=5) bones

freezer at -80℃.

Some cores were damaged during drilling or not available due to the vertebrae were too small (n=21).

Expected cylindrical cores (**n= 9)**.

1 core was excluded due to an error in the drilling direction.

Actual cylindrical cores (**n=8**) from left and right sites.

Actual cylindrical cores (**n=54**) from left, middle, and right sites.

1 sample can be obtained from one cylindrical core (length 15-20mm).

3 samples can be obtained from one cylindrical core (length about 42mm).

Expected samples (**n=24**) from left and right sites.

Expected samples (**n=54**) from left, middle, and right sites.

9 samples were added due to longer (about 26mm) cylindrical cores.

1 sample was damaged during the cutting.

Actual samples (**n= 23)** from left and right sites.

Actual samples (**n=63**) from left, middle, and right sites.

Saline and acetone-treated group (**n=18**)

Saline-immersed group (**n=35**)

Middle site (**n=19**)

Middle site (**n=21**)

Left site (**n=12**)

Right site (**n=15**)

Left site (**n=4**)

Right site (**n=3**)

A part of samples was excluded due to non-compliance with inclusion criteria and methodological errors, the final numbers used for statistical analysis are as follows.

Right site (**n=14**)

Left site (**n=9**)

Right site (**n=24**)

Left site (**n=18**)

Supplement 1. Flow chart of the detail data on the samples tested in our study.
