# Supplementary material for: Selected mechanical properties of human cancellous bone subjected to different treatments: short-term immersion in physiological saline and acetone treatment with subsequent immersion in physiological saline
Source: J Orthop Surg Res. 2022 Aug 6;17:376. doi: 10.1186/s13018-022-03265-4 (PMC9357305; doi:10.1186/s13018-022-03265-4)
Supplement: Supplementary file 2 — Additional file 2. The detailed data of donor bones, cylindrical cores, and samples in our study. [file 13018_2022_3265_MOESM2_ESM.docx]

| Bones | Expected donor bones | Actual donor bones | Expected cores | Actual cores | ^a^Expected samples | ^b^Actual samples  (Total) | Actual samples (L) | Actual samples (M) | Actual samples (L+M) | Actual samples (R) | ^c^Final samples (Total) | Final samples (L) | Final samples (M) | Final  samples (L+M) | Final samples (R) |
| --- | --- | --- | --- | --- | --- | --- | --- | --- | --- | --- | --- | --- | --- | --- | --- |
| T_12_ | 5 | 1 | 3 | 2 | 2 | 2 | 1 | 0 | 1 | 1 | 1 | 0 | 0 | 0 | 1 |
| L_1-5_ | 25 | 19 | 57 | 45 | ^a^45 | ^b^54 | 15 | 19 | 34 | 20 | 40 | 11 | 17 | 28 | 12 |
| S_1-5_ | 5 | 5 | 15 | 7 | 7 | 7 | 2 | 2 | 4 | 3 | 4 | 1 | 2 | 3 | 2 |
| **Total vertebrae** | **35** | **25** | **75** | **54** | **54** | **63** | **18** | **21** | **39** | **24** | **45** | **12** | **19** | **31** | **15** |
| L_F | 5 | ^d^4 | 4 | ^e^3 | 9 | 9 | 9 | 0 | 9 | 0 | 4 | 4 | 0 | 4 | 0 |
| R_F | 5 | 5 | 5 | 5 | 15 | ^f^14 | 0 | 0 | 0 | 14 | 3 | 0 | 0 | 0 | 3 |
| **Total femoral heads** | **10** | **9** | **9** | **8** | **24** | **23** | **9** | **0** | **9** | **14** | **7** | **4** | **0** | **4** | **3** |
| **Total bones** | **45** | **34** | **84** | **62** | **78** | **86** | **27** | **21** | **48** | **38** | **52** | **16** | **19** | **35** | **18** |

Supplement 2. The detailed data of donor bones, cylindrical cores, and samples in our study.

Note: T_12_ (thoracic vertebral body), L_1-5_ (lumbar vertebral bodies), S_1-5_ (sacral bones), L_F (left femoral head), R_F (right femoral head). ^a^Expected samples: the samples (Ø6 ×12 mm) collected from actual cores, of theses, the most of cylindrical cores from lumbar vertebral bodies were about 15-20 mm, and 1 sample can be obtained, but 9 of them were about 26 mm, and 2 samples can be obtained. Thus, ^b^Actual samples (Total) obtained from the lumbar vertebral bodies were 9 more than ^a^Expected samples. ^c^Final samples: the stress-strain curves after testing were checked, and samples that did not meet the inclusion criteria as well as those due to methodological errors were excluded, the remaining samples were used for the final statistical analysis. ^d^Actual L_F: one of the donor bones was the presence of a prosthesis in the left hip, so the femoral head cannot be used. ^e^L_F: one cylindrical core was excluded due to a deviation in the drilling direction. ^f^R_F: one sample was damaged during the cutting.
